# Why Age Matters in Higher Education

**DOI:** 10.1093/geroni/igaa057.2088

**Published:** 2020-12-16

**Authors:** Joann Montepare

**Affiliations:** Lasell University, Newton, Massachusetts, United States

## Abstract

At the core of behavioral and social scientists’ work in the aging field is informing and supporting the well-being of individuals and their communities. With the shift in age demographics and the aging of our populations, broadening educational efforts are more important than ever. However, advancing knowledge about aging and creating age inclusive educational opportunities has been a challenge in higher education, reflecting its historical, age-segregated structure, among other things. The pioneering Age-Friendly University (AFU) initiative, recently endorsed by GSA’s Academy for Gerontology in Higher Education (AGHE), offers a valuable set of guiding principles that institutions in higher education can use to assess the extent to which their programs and practices are age inclusive, as well as identify gaps and opportunities. This presentation will discuss how the time has come for scientists to help shape more age-friendly institutions, and what they can look like in the years to come.

